# Toxoplasmosis Infection during Pregnancy

**DOI:** 10.3390/tropicalmed8010003

**Published:** 2022-12-21

**Authors:** Myla Deganich, Crystal Boudreaux, Imaan Benmerzouga

**Affiliations:** 1 West Virginia School of Osteopathic Medicine, Lewisburg, WV 24901, USA; 2 Kiran C. Patel College of Osteopathic Medicine, Nova Southeastern University, Clearwater, FL 33759, USA

**Keywords:** congenital toxoplasmosis, infection, pregnancy, *Toxoplasma gondii*

## Abstract

This literature review aims to give an overview of the current knowledge concerning how a toxoplasmosis infection affects the mother and her fetus. A thorough search of PubMed and a complimentary search of Google Scholar databases were used to identify relevant studies for this review. Although a *Toxoplasma gondii* infection is preventable, this infection is contracted by consuming contaminated food and water and by exposure to environmental sources of infection such as contaminated soil. Maternal-to-fetal transmission of this infection can result in devastating ophthalmic and neurological consequences for the fetus. Although a toxoplasmosis infection can result in long-term effects on the fetus, chronic disease is also associated with mental illness in mothers. Effective treatment can reduce the risk of congenital toxoplasmosis and the long-term consequences of infection in the fetus. Without appropriate screening and education programs, this infection will remain largely undiagnosed.

## 1. Introduction

*Toxoplasma gondii* (*T. gondii*) causes the disease toxoplasmosis. In the United States, *T. gondii* infects approximately 1.1 million people yearly [1], with 789 deaths associated with toxoplasmosis between 2000 and 2010 [2]. If this infection is acquired during pregnancy, it could result in congenital toxoplasmosis. Clinical manifestations of infection can also be seen later in infancy, childhood, or adolescence [3]. Among those infected individuals, toxoplasmic chorioretinitis occurs in approximately 21,000 people annually. Fortunately, the rates of infected individuals in the United States have been declining. According to the National Health and Nutrition Examination Survey (NHANES), the number of infected women has decreased, with a seroprevalence rate of 6% for women born in the United States during 2009–2010. However, among these women, African Americans and Hispanic Americans have the most increased mortality associated with infection [2]. There has also been a decrease in the seroprevalence among women born outside of the United States. From 1988–1994, the seroprevalence of infected women aged 15 to 44 was 15%, whereas in 1999–2004, this seroprevalence decreased to 11%, and in 2009–2010, the seroprevalence was 9.1% [4]. Additionally, a study analyzing the global rates of latent toxoplasmosis infection in pregnant women of varying ages found the age groups with the lowest and highest rates of latent infection were those less than twenty years old and those greater than thirty-six years old, respectively [5]. Furthermore, another study which examined the global rate of acute toxoplasmosis infection in women found an overall prevalence of 1.1% [6].

This literature review summarizes the current literature on the short-term and long-term effects of Toxoplasma on the fetus, and the effect of infection on the mother. As other literature reviews provide limited information on these topics, this review summarizes the current knowledge on these topics.

## 2. Materials and Methods

A thorough search of PubMed and a complimentary search of the Google Scholar databases were completed to identify studies for this literature review. Although there was no restriction on the publication year, searches were limited to those available in the English language. Studies were then assessed to determine their relevance for meeting the following objectives: risk factors and routes of infection, life cycle and mechanism of infection, symptoms of infection, effects of infection on the fetus, long-term effects of infection on the fetus and mother, treatments, and prevention strategies.

## 3. Toxoplasmosis Pathobiology

Toxoplasma is a widespread parasite and, as a result, many risk factors for infection have been identified including working with meat, eating raw or undercooked meat, having contact with cat feces, and drinking unfiltered water or unpasteurized goat milk [7,8]. Similarly, other sources of exposure are lakes, reservoirs, and contaminated soil. Although meat is not typically eaten undercooked, most infections throughout the United States are from eating undercooked meat [7,9,10]. Infection can also occur via mother-to-fetus transmission through the placenta, via an organ transplantation from an infected donor [11,12], through a blood transfusion, or through a laboratory accident [13], albeit these are rare. Finally, traveling to countries with a high prevalence of *T. gondii*, especially those in which the more virulent genotypes are present, is a significant risk factor for infection [10,14]. However, up to 50% of people diagnosed with a *T. gondii* infection could not identify a known risk factor [15]. *T. gondii* can exist in three forms: a tachyzoite, a bradyzoite, or a sporozoite [16]. Tachyzoites are the fast replicating forms of *T. gondii,* whereas bradyzoites are the dormant form and are present within tissue as cysts [17]. Sporozoites are the infectious forms of *T. gondii* which are dormant in the environment [17], and due to their stability, can remain infectious for many years despite being exposed to standard disinfectants, and hot, cold, dry, or moist environments [18].

Toxoplasma has a sexual and asexual life cycle. The sexual life cycle occurs in the feline and begins when the parasite is ingested. Once ingested, the parasite enters the small intestine’s epithelial cells and undergoes multiple stages of development. After, the parasite is returned to the environment in the feline’s feces, where it undergoes sporulation, producing an oocyst. The asexual life cycle begins once an intermediate host ingests this oocyst. Once ingested, the small intestine epithelial cells are infected, and tachyzoites are produced [19], which indicates an active infection [17]. The tachyzoites then move through the blood and lymphatics to target organs such as nervous tissue, muscle, and visceral organs. Once the target organ is reached, replication occurs, and Toxoplasma will lay dormant as bradyzoites.

## 4. Toxoplasmosis Infection during Pregnancy

If infection occurs during pregnancy, it is transferred from a mother to her fetus through the placenta [20]. The transmission risk depends on the gestational age at which the infection occurred. The transmission risk increases with increasing gestational age; however, the disease severity decreases as the gestational age increases [3]. Additionally, other factors, such as immune factors, the virulence of *T. gondii* strains, and differences in genotypes, may also affect disease severity [3,21,22]. Although this infection is typically transferred from a mother to her fetus if acquired during pregnancy [20], a chronically infected mother can transfer this infection to her fetus if her infection is reactivated during her pregnancy. A prior infection can be reactivated if the mother is severely immunocompromised [23]. Additionally, a previously immunocompetent mother can become immunocompromised if treated with glucocorticoids for an underlying disease [20]. In those who are immunocompromised or immunosuppressed, bradyzoites can be released from cysts and converted back to tachyzoites, which causes the reactivation of toxoplasmosis and, as a result, can present in the fetus as encephalitis or chorioretinitis [17]. Most infected women do not show symptoms [15,20]. In a study of 76 women who had given birth to congenitally infected children, 52% were asymptomatic [15,20]. However, if symptoms do occur, they usually develop 5 to 23 days after the infection [24] and are typically nonspecific and mild [20,25], including chills, fever, headache, sweats, sore throat, lymphadenopathy, hepatosplenomegaly, maculopapular rash, and myalgias [20,25]. The most common symptom is lymphadenopathy, as reported in 7% of pregnant women in a prospective European study [26]. Although the fever typically lasts for 2 to 3 days [20,25], the lymphadenopathy can last for weeks [26]. In rare situations, women can have vision changes, which is due to chorioretinitis either from their acutely acquired infection or from the reactivation of a chronic infection [27]. Interestingly, most infected women are not exposed to the typical risk factors for infection [15]. In the previously mentioned study, 61% were not exposed to raw meat or cat litter [15,20]. Thus, it is necessary to identify additional risk factors that have yet to be recognized.

## 5. Global Burden of Congenital Toxoplasmosis

Toxoplasma’s ability to infect a wide variety of hosts in addition to the many risk factors for infection makes this parasite a global health concern, with an approximate infection rate of up to one third of the global population [28]. As a result, congenital toxoplasmosis remains a considerable cause of morbidity and mortality throughout the world, especially in developing countries. A study examining the incidence of congenital toxoplasmosis estimated 190,000 cases annually, which represented a rate of roughly 1.5 cases of congenital toxoplasmosis per every 1000 live births [29].

## 6. Congenital Toxoplasmosis and Treatment

### 6.1. Outcomes of Toxoplasmosis Treatment during Pregnancy

The effects of infection on the fetus can be examined by comparing the consequences of maternal treatment as compared to no treatment. When treating congenitally infected infants, the preferred regimen is pyrimethamine plus sulfadiazine and folinic acid [30,31]. Treatment is most effective at approximately less than three weeks from seroconversion, as treatment is not effective once toxoplasma converts to the dormant form. If mothers are treated with pyrimethamine-sulfadiazine after seroconversion, studies suggest there is a decreased risk of congenital infection [32]; however, there is some variability in the literature as to which treatment, pyrimethamine-sulfadiazine or spiramycin, is more effective in reducing the risk of congenital toxoplasmosis.

In a randomized study of 143 infected mothers, there was a lower transmission rate of congenital disease in those treated with sulfadiazine plus pyrimethamine (18.5%) as compared to those treated with spiramycin (30%) [32]. A study from Italy also showed mothers treated with pyrimethamine-sulfonamide showed a decreased maternal-to-fetal transmission rate compared to spiramycin alone [33]. However, this result could have been due to the differences in screening for infection between mothers. In another European study examining the maternal-to-fetal transmission rate of infection based on gestational age and treatment, women who were infected during the 12th week of gestation transmitted the infection to their fetus 9% of the time as compared to the 83% in those infected at 40 weeks of gestation [26]. If an infection is acquired early in pregnancy and treatment is not given, severe consequences, such as fetal death, are likely [3]. If death does not occur, the fetus can develop severe symptomatic infections that can localize to different organs or which may be more generalized [30] (e.g., these infections may range from involving the eyes to the central nervous system (CNS) [3]).

### 6.2. Outcomes of Toxoplasmosis Treatment in Congenitally Infected Infants

Some studies suggest treatment will also improve the adverse sequelae of congenital infection. In a study of 120 congenitally infected infants, infants were treated with a dose of sulfadiazine once a day, folinic acid three times a week, pyrimethamine once a day for two or six months, which was then followed by pyrimethamine three times a week for the rest of the year, for a total of 12 months [30]. When comparing treated infants with the control of untreated infants, those who were treated had improved neurologic, cognitive, ocular, and auditory outcomes [30]. All treated infants had normal cognitive, neurologic, and auditory symptoms. Finally, 72% of infants with a moderate to severe illness at birth had normal neurologic and cognitive symptoms after treatment. 

In a European study that followed a cohort of 293 congenitally infected infants who received prenatal treatment, the risk of death or developing severe neurological symptoms was decreased by approximately 75% [34]. Additionally, the infants of mothers treated during pregnancy had a 25.7% chance of either dying or developing severe neurological symptoms as compared to the 60% chance of these events occurring without treatment. However, as there was uncertainty about when maternal seroconversion occurred, this study should be viewed cautiously. Although studies show improved symptoms with treatment [30,35], the medications used to treat congenital infection have side effects. The main adverse effect of pyrimethamine is neutropenia [36]. Other possible side effects include hepatotoxicity, aplastic anemia, leukopenia, dose-related bone marrow suppression, thrombocytopenia, and a hypersensitivity reaction. Sulfadiazine can cause allergic dermatitis, hives, and neutropenia. Nonetheless, if no treatment is given, the risk of developing long-term sequelae, such as neurologic abnormalities, psychomotor and mental disabilities, and chorioretinal disease, is much higher. For example, without treatment, chorioretinal disease develops in up to 85% of congenitally infected children [20]. Manifestations of congenital toxoplasmosis can vary from severe, mild, to moderate, and can be subclinical or be seen clinically later in infancy, childhood, or adolescence [3]. In a study conducted in France, one-third of children showed severe symptoms from their disease, such as macular retinochoroiditis, hydrocephalus, or having a disseminated form of the disease. In contrast, two-thirds of newborns showed moderate symptoms such as peripheral retinochoroiditis or intracranial calcifications. Finally, roughly 90 percent of children who were born with congenital toxoplasmosis were asymptomatic at birth [37]. In a separate study, 70 to 90 percent of newborns with congenital toxoplasmosis did not show any indications or symptoms of infection during a routine physical examination, and 10 to 30 percent of congenitally infected infants displayed clinical manifestations and symptoms at birth or early in their infancy [38,39].

### 6.3. Outcomes of Untreated Congenital Toxoplasmosis

Untreated newborns who have a mild or subclinical form of the disease at birth have an increased risk of complications [40]. Although the most common late complication of congenital toxoplasmosis is chorioretinitis, other possible complications are microcephaly, seizures, sensorineural hearing loss, motor dysfunction, cerebellar dysfunction [40], slowed growth, and endocrine abnormalities [41,42]. Additionally, chorioretinitis has associated symptoms such as retinal detachment, loss of vision, cataracts, glaucoma, and changes in the iris, with new onset retinal lesions occurring most frequently in late childhood and adolescence [43]. Collectively, these data highlight the importance of treating an infected mother as well as treating congenital toxoplasmosis in infants, which would be made possible with prenatal screening.

## 7. Long-Term Effects of Toxoplasmosis

Early intervention during pregnancy reduces the adverse consequences of a congenital infection; however, even with treatment, infants are still at risk for developing sequelae later in life. Furthermore, there is a continued risk of developing adverse sequelae because the medications used to treat the congenital infection do not kill bradyzoites. Thus, there is the possibility of reactivation, especially in the heart and central nervous system [31,44,45,46,47]. If treatment is administered in utero or within the first two months after birth and continued for at least one year, then adverse neurologic complications are uncommon. However, late neurologic manifestations may still occur in rare instances. Table 1 and Table 2 summarize the outcomes of treatment as compared to no treatment.

These studies highlight the need for additional long-term studies to understand the long-term effects of prenatal toxoplasmosis infection and the impact of treatment on these effects.

## 8. Prevention

Prevention is the primary method to reduce the risk of infection with toxoplasma. The primary prevention strategy involves providing educational materials on possible preventative measures, and should be integrated into prenatal visits, classes, and programs. The use of educational materials can decrease seropositivity rates [9,55]. Among primary preventive efforts, women should avoid sources of infection, not drink unfiltered water [10,56], and maintain hand hygiene. Thoroughly washing fruits and vegetables is another way to avoid infection [9,14]. As stated previously, most infections in the United States are from undercooked meat [7,9,10]. Therefore, caution should be taken when preparing meat. When cooking meat, it takes one hour at 50 °C to inactivate tissue cysts; however, when the internal temperature reaches 67 °C, the tissue cyst is immediately killed [57]. Using a high pressure of 300 MPa can also inactivate tissue cysts [58]. Freezing meat to an internal temperature of 10 °F [−12 °C] or less can also kill tissue cysts [59]. However, using a microwave is not effective in killing tissue cysts [60]. Women should also thoroughly wash counters, sinks, cutting boards, and knives after meal preparation [9,10,14,56]. There is also some evidence that smoked or cured meat is unsafe, with an increased risk of infection when cured meat products consist of multiple animal sources [10,61,62]. Interestingly, cat ownership is not highly associated with infection. Nevertheless, women should still take caution in cleaning litter boxes to avoid accidental exposure, or another person should clean the litter box instead [9,10,14]. As cats primarily become infected by hunting or eating raw meat [63], they can be kept indoors to decrease infection risk [64]. However, the correlation between indoor cats and infection risk has not been directly studied. A vaccine for cats may also decrease the number of oocysts shed. The attenuated T-263 strain, a live vaccine, was experimentally injected into infected kittens, which prevented 31 out of the 37 kittens from shedding oocysts [65]. Nonetheless, this vaccine has disadvantages: its shelf life is limited, it requires cold chain administration, and it has the risk of being hazardous to the person administering the vaccine [66]. Screening as a secondary prevention is essential to identify infected women early. Several European observational studies have shown a decreased risk of maternal–fetal transmission after national prenatal maternal screening, fetal diagnosis, and treatment programs were implemented [67,68,69]. However, until screening programs are implemented and educational programs become more effective and widespread, congenital toxoplasmosis will remain undiagnosed [20]. Finally, if a woman becomes infected before pregnancy, pregnancy should be delayed for at least one to three months, making maternal-to-fetal transmission less likely [70]. However, there is limited evidence to base a recommended time to postpone pregnancy for after infection.

## 9. Conclusions

Although a toxoplasmosis infection is preventable, if contracted during pregnancy, this infection can result in devastating consequences for the fetus. In addition to the long-term adverse effects on the fetus, chronic disease is also associated with mental illness in mothers. Preventative measures are necessary to avoid infection in seronegative women. However, this infection will remain largely undiagnosed and untreated without appropriate educational and screening programs. Effective treatment can reduce the risk of congenital toxoplasmosis and the long-term consequences of the infection on the fetus. State-wide educational programs and cost-effective screening programs for pregnant women should be implemented to reduce this disease’s emotional and financial burden.

## Figures and Tables

**Table 1:** Summary of studies treating toxoplasmosis.

Reference	No.	Observation Period (Years)	Finding(s)
Wallon, 2014 [47]	477	10.5	Two percent of the patients developed severe neurological abnormalities, whereas 30 percent of the treated patients developed chorioretinitis at a median age of 3.1 years. However, most of the patients in this study who had developed chorioretinitis had normal vision, with 81 percent developing unilateral lesions and 73 percent developing bilateral lesions. Less than 2 percent of these patients developed deterioration in their vision.
Berrébi, 2010 [31]	666	20	A total of 112 (17%) infants who were born to mothers treated with either spiramycin alone or in combination with pyrimethamine-sulfadoxine were diagnosed with congenital toxoplasmosis. Among the children diagnosed with congenital toxoplasmosis, 79 children did not have symptoms of their congenital infection (74%), 28 children had chorioretinitis (26%), and one child had serious neurological symptoms.
Peyron, 2011 [46]	126	Not reported	In total, 11.8% (12 patients) exhibited neurological symptoms of their infection, whereas 58.8% (60 patients) developed ocular lesions, and 12.7% (13 patients) had reduced visual function. However, the overall quality of life score was close to the general population (74.7 ± 14.2 as compared to 73.7 ± 15.3), and their visual ability was only slightly impaired.
Vishnevskia-Dai, 2020 [48]	22	2007–2016	The ocular lesion initially reduced in size; however, there was a limited reduction in the size of the lesion thereafter.
Lago, 2021 [49]	77	1996–2017	A lesser amount of new retinochoroidal lesions developed in patients who received treatment earlier on in the infection; 33.3% of patients who were treated before 2 months old developed these lesions, whereas those treated before 4 months old developed these lesions 77.8% of the time.
Fernandes, 2020 [50]	141	1	Among the patients treated with a placebo as compared to trimethoprim-sulfamethoxazole for their unilateral retinochoroiditis, there was a significantly greater chance of recurrence during the 6-year long follow-up.
McLeod, 2006 [30]	120	1981–2004	Infants without severe neurological impairment who were treated with pyrimethamine and sulfadiazine had normal cognitive and auditory outcomes, whereas the treatment of infants with moderate to severe neurological impairment resulted in normal neurological outcomes in more than 72% of patients.

**Table 2:** Summary of studies where toxoplasmosis was not treated.

Reference	No.	Observation Period (Years)	Finding(s)
Saxon, 1973 [51]	16	Not reported	The subclinical/untreated children had a significantly lower mean intelligence quotient (IQ) compared to the uninfected children.
Phan, 2008 [43]	38	1981–2005	Roughly 90 percent of infected children developed new-onset chorioretinitis lesions without treatment, with this risk continuing into adulthood.
Pedersen, 2011 [52]	45,609	1992–2008	This study found a correlation between the levels of *T. gondii* IgG antibodies and schizophrenia, with a higher risk being associated with increasing antibody levels.
Pedersen, 2021 [53]	45,788	1992–2006	This study found that infected mothers had an increased risk of self-directed harm compared to uninfected mothers, with a higher risk being associated with increasing antibody levels. Additionally, the risk of attempting suicide and a successful suicide were 1.81 and 2.05, respectively, suggesting an increased risk of self-directed injury among infected women.
Gao, 2019 [54]	475	Not reported	This study did not find an association between infection and postpartum depression; however, the small sample size of this study may have skewed this result.

## Data Availability

Not applicable.
